# Biosurfactant production from marine bacteria associated with sponge *Callyspongia diffusa*

**DOI:** 10.1007/s13205-014-0242-9

**Published:** 2014-08-13

**Authors:** Asha Dhasayan, Joseph Selvin, Seghal Kiran

**Affiliations:** 1 Department of Microbiology, School of Life Sciences, Bharathidasan University, Tiruchirappalli, 620024 Tamil Nadu India; 2 Department of Microbiology, Pondicherry University, Puducherry, 605014 Tamil Nadu India; 3 Department of Food Science and Technology, Pondicherry University, Puducherry, 605014 Tamil Nadu India

**Keywords:** Marine sponge, *Callyspongia diffusa*, Biosurfactant, Sponge-associated bacteria, *Bacillus amyloliquefaciens*, Lipopeptide

## Abstract

Marine-derived biosurfactants have gained significant attention due to their structural and functional diversity. Biosurfactant production was performed using bacteria associated with *Callyspongia diffusa*, a marine sponge inhabiting the southern coast of India. A total of 101 sponge-associated bacteria were isolated on different media, of which 29 isolates showed positive result for biosurfactant production. Among the 29 positive isolates, four were selected based on highest emusification activity and were identified based on 16S rDNA sequence analysis. These isolates were identified as *Bacillus subtilis* MB-7, *Bacillus amyloliquefaciens* MB-101, *Halomonas* sp. MB-30 and *Alcaligenes* sp. MB-I9. The 16S rDNA nucleotide sequences were deposited in GenBank with accession numbers KF493730, KJ540939, KJ414418 and KJ540940, respectively. Based on the highest oil displacement activity and effective surface tension reduction potential, the isolate *B. amyloliquefaciens* MB-101 was selected for further optimization and structural delineation. The production of biosurfactant by the isolate was significantly enhanced up to 6.76 g/l with optimal concentration values of 2.83 % for glycerol, 2.65 % for peptone, 20.11 mM for ferrous sulfate and 74 h of incubation by employing factorial design. The structural features of the purified biosurfactant from *B. amyloliquefaciens* MB-101 showed similarity with lipopeptide class of biosurfactant. In conclusion, the present study emphasizes the utilization of marine sponge-associated bacteria for the production of biosurfactant that may find various applications.

**Electronic supplementary material:**

The online version of this article (doi:10.1007/s13205-014-0242-9) contains supplementary material, which is available to authorized users.

## Introduction

In recent years, explorations for effective biosurfactants have been increasing to satisfy their demand in wider industrial and environmental applications (Sachdev and Cameotra 2013). Biosurfactants are surface active compounds having both hydrophilic and hydrophobic domain that allows them to exist preferentially at the interface between polar and non-polar media, thereby reducing surface and interface tension (Banat et al. 2010). These molecules comprise complex structures which are grouped either as low (glycolipids and lipopeptides) or high (polymeric biosurfactants) molecular weight compounds (Cameotra et al. 2010). During the last decade, biosurfactants have been used as alternatives for synthetic surfactants and are expected to find many industrial and environmental applications such as enhanced oil recovery, crude oil drilling, lubrication, bioremediation of pollutants, foaming, detergency, wetting, dispersion and solubilization (Singh et al. 2006). The application of biosurfactant also increased in cosmetic, health care and food processing industries (Makkar and Cameotra 2002). Biosurfactants display important biological activities including antimicrobial, insecticidal, immune-modulative and antitumoral activities (Cao et al. 2009; Liang et al. 2014).

Potent biosurfactant producing microorganism from marine environment demands reduced utilization of synthetic surfactants and probably favors the increased use of easily biodegradable and environmentally benign biosurfactant molecules (Das and Mukherjee 2007). Marine sponges are considered as one of the marine environmental niche, harboring diverse microbial communities with considerable ecological and biotechnological importance. Since sponges are filter feeders, they consume microorganisms from the surrounding seawater. Any microorganism that resists the digestive process and immune response of the sponge can successfully maintain symbiotic relationship with sponges. These microbial communities involve sponge specific bacteria, archaea and eukaryotic microorganisms which do not inhabit the surrounding marine environment but live exclusively within sponge hosts (Hentschel et al. 2002). Thus, marine sponge-associated microorganisms have drawn an immense attention as a source for new secondary metabolites, because of their wider biochemical accessibility, stability and higher activity than terrestrial counterparts (Skariyachan et al. 2014; Kiran et al. 2014b).

The sponge-associated bacteria for the biosurfactant production are scarcely reported. Culturable marine bacteria from marine sponge may act as novel sources of lipopeptide and glycolipid biosurfactants (Kiran et al. 2010). Thus, the present study was initiated to isolate sponge-associated bacteria and explore them as a sustainable source for the production of novel biosurfactants. A total of 101 isolates were obtained from the marine sponge, *Callyspongia diffusa*. Among the 29 biosurfactant producers, four isolates viz, *Bacillus subtilis* MB-7, *Bacillus amyloliquefaciens* MB-101, *Halomonas* sp. MB-30 and *Alcaligenes* sp. MB-I9, exhibited highest biosurfactant producing potential. Particularly, the isolate, *B. amyloliquefaciens* MB-101, showed effective oil displacement and surface tension reduction potential. Hence it was selected for further optimization and structural characterization.

## Materials and methods

### Chemicals

All the chemicals and media components used in the experiments were purchased from HiMedia, Genei and Sigma and were of analytical grade.

### Collection and processing of marine sponge specimen

Marine sponge (*C. diffusa*) was collected from a depth of 10–15 m, growing at the southeast coast of Kanyakumari (8°04′46″N × 78°13′41″E), southern India. The collection site comprises intertidal rocky substratum and mineral rich sand. The collected specimens were kept in separate sterile bags, transferred to the laboratory on ice and further processed according to the protocol by Selvin et al. (2004). Only unbroken samples were used for microbiological analysis to avoid cross contamination. The specimens were kept aside for 2 h in sterilized aged seawater to eliminate non-associated bacteria from inner and outer surfaces of the host sponge by digestion. Environmental water representing the sponge habitat was taken prior to sponge sampling and filled up in sterile glass bottles.

### Isolation of sponge**-**associated bacteria

Sponge tissue (1 cm^3^) was excised from the middle of the intact sponge under aseptic conditions and washed thrice with sterile seawater to remove any bacteria within the current canals. A tissue homogenizer was used to homogenate the sponge tissue uniformly. The resultant homogenate was serially diluted using sterilized aged seawater and plated on nutritive media (Gandhimathi et al. 2008). The isolation was performed on various media such as marine sponge agar (MSA: raffinose, 10 g; l-histidine, 1 g; ferrous sulphate, 0.01 g; dipotassium hydrogen phosphate, 1 g; calcium carbonate, 0.5 g; agar, 15 g; sodium chloride, 20 g; aqueous host sponge extract: 100 ml; double distilled water, 900 ml; pH 7.8; autoclaved at 15 lbs for 15 min), ZoBell marine agar (ZMA: peptone, 5 g; yeast extract, 1 g; FePO_4_.4H_2_O, 0.01 g; agar 15 g; aged seawater, 750 ml; distilled water, 250 ml; pH 7.2), Emerson agar (EA: Beef extract, 4 g; yeast extract, 1 g; peptone, 4 g; dextrose, 10 g; sodium chloride, 2.5 g; agar, 20 g; pH 7.2), sea water agar (yeast extract, 5 g; peptone, 5 g; beef extract, 3 g; sodium chloride, 24 g; potassium chloride, 0.7 g; magnesium chloride, 5.3 g; magnesium sulphate, 7 g; calcium chloride, 0.1 g; pH 7.5) and nutrient agar supplemented with 2.5 % NaCl. Amphotericin B (30 g/l) was added to all media to inhibit fungal growth. Plates were incubated at 30 °C for 7 days under dark condition. Following incubation, morphologically distinct bacterial colonies were selected, cultured on ZMA and stored at 4 °C until use.

### Screening assays for biosurfactant production

To analyze their potency to produce biosurfactant, the isolates were tested for lipase activity, hemolytic activity, oil-spreading test and drop collapse test. Lipase activity was screened on tributyrin agar plates. Hemolytic activity was performed on blood agar plates containing sheep blood (5 % v/v) (Carrillo et al. 1996).

Biosurfactant production was examined with drop collapsing test according to the protocol given by Youssef et al. (2004). The culture supernatant that made the drop to collapse was indicated ‘positive’ and the drop that remained beaded was scored ‘negative’. Oil displacement test was determined according to the protocol given by Morikawa et al. (1993). Extracellular anionic biosurfactant producers were screened by blue agar plates containing cetyltrimethylammonium bromide (CTAB) (0.2 mg/ml) and methylene blue (5 µg/ml) (Siegmund and Wagner 1991). Isolates showed high biosurfactant activity were inoculated on ZMB supplemented with 1 % glucose and incubated under shake flask condition to examine the surface tension reduction and emulsification activity (Joshi et al. 2008). Bacterial adhesion to hydrocarbon (BATH) assay was also performed for screening potent biosurfactant producers (Pijanowska et al. 2007). All the assays were performed in triplicates.

### Emulsification activity

Kerosene was added to cell-free broth in a ratio of 1:1 and vortexed vigorously for 2 min. Sterile distilled water was used as the negative control (Paraszkiewicz et al. 1992). After 24 h of incubation at 30 °C, the emulsification index (*E*_24_) was estimated using a formula: *E*_24_ = (*H*_EL_/*H*_S_) × 100, where, *E*_24_ is the emulsification index after 24 h, *H*_EL_ is the height of the emulsified layer and *H*_S_ is the height of the total liquid column.

### Biochemical and molecular characterization of biosurfactant producers

The morphological and biochemical characterization of four selected biosurfactant producing bacteria were performed according to Bergey’s manual of determinative bacteriology, 9th edition (Holt et al. 1994). For molecular identification, genomic DNA was isolated (Enkicknap et al. 2006) and 16S rDNA sequences were amplified by polymerase chain reaction (PCR). PCR was carried out on a master cycler gradient (Eppendorf), in a 50 μl reaction mixture. The reaction mixture contained 5 μl amplification buffer (10×), 5 μl MgCl_2_ (1.5 mM), 3 μl of 10 pM forward primer (5′-AGAGTTTGATCMTGGCTCAG-3′), 3 μl of 10 pM reverse primer (5′-AAGGAGGTGATCCAGCC-3′), 1 μl dNTPs and 0.25 μl Taq DNA polymerase. The thermocycler was programmed as follows: 1 min initial denaturation at 95 °C, followed by 35 cycles that consisted of denaturation for 45 s at 94 °C, annealing for 30 s at 55 °C and extension at 72 °C for 2 min followed by a final extension of 8 min at 72 °C.

The resulting PCR products were sequenced and the 16S rDNA sequences obtained from the bacterial isolates were compared with known 16S ribosomal sequences in NCBI database using BLASTn (Altschul et al. 1990). Multiple sequence alignments were carried out using ClustalW and phylogenetic tree was constructed using neighbor-joining (NJ) algorithm available in Molecular Evolutionary Genetics Analysis software version 6.0 (http://www.megasoftware.net). The nucleotide sequences were submitted to GenBank database (NCBI, USA).

### Statistical optimization of biosurfactant production by *B. amyloliquefaciens* MB-101

In this study, maximum biosurfactant production by *B. amyloliquefaciens* MB-101 was attained by response surface statistical optimization methods employing different process parameters under submerged fermentation. Significance of various medium constituents toward biosurfactant production was tested initially by a fractional factorial experimental design. The factors and ranges were selected by one-factor experiments (data not shown). The 2^9−5^ fractional factorial design consisted of a set of 16 experimental runs in which the selected nine factors were kept either at their high (+) or low (−) levels to find out the most significant factors on biosurfactant production (Online resource 1, 2). All these experiments were carried out in 200 ml Erlenmeyer flasks containing 50 ml of production medium with appropriate media components. The minimal medium (g/l) containing yeast extract 0.05; KH_2_PO_4_ 0.2; K_2_HPO_4_ 0.2; NH_4_Cl 0.25; KCl 0.5; FeSO_4_.6H_2_O 0.1; FeCl_3_.6H_2_O 0.1; MgCl_2_.6H_2_O 0.6; Na_2_SO_4_ 2.84; trace elements (1 ml/l) supplemented with different carbon, nitrogen and metal ions at appropriate concentrations predicted by fractional factorial design were used for statistical optimization. These statistically designed media were inoculated with the seed culture at 2.5 % (v/v) and incubated at 37 °C for 96 h. At the end of the fermentation, biosurfactant concentration in the cell-free medium was estimated by gravimetric method after acid precipitation and solvent extraction and also by calculating emulsification activity. All experiments were performed in triplicate and at two different occasions and the responses considered for analysis represent mean of these responses. Based on the first-order model equation obtained by the fractional factorial design, a series of trials were performed in the direction of the steepest ascent. To fit empiric second-order polynomial model, a central composite design (CCD) with 5 coded levels was performed. A 2^4^ full factorial CCD for four test variables (glycerol, peptone, ferrous sulphate and incubation time) each at five levels with six replicates at the centre points was employed to fit a quadratic model, indicating that 30 experiments were required for the procedure. The model determined an analysis of variant (ANOVA) statistically from the regression model developed from the responses, and the *F* and *R* ^*2*^ (determinant coefficient) values were evaluated for generating contour plots and three dimensional response surface graphs. These plots were used to understand the interaction of different factors and to predict optimum medium composition for biosurfactant production. The optimal values of the experimental conditions were obtained by solving the regression equation and analyzing the response–surface contour plots. All statistical analyses were carried out by Design-Expert software package (version 9.0.0.7, Stat-Ease, Inc., USA).

### Structural characterization

The biosurfactant from the culture supernatant of *B. amyloliquefaciens* MB-101 was separated by acid precipitation followed by solvent extraction (Vater et al. 2002). Briefly, the seed culture of biosurfactant producing bacteria MB-101 was prepared and transferred to a 500 ml of optimized nutritive medium containing glycerol 2.84 %; peptone 2.65 %; FeSO_4_.6H_2_O 20.11 mM, NaCl 2 % with other minimal media components and 1 % glucose. Submerged fermentation in shake flask was developed in the buffered medium at pH 7 and incubated at 37 °C in 150 rpm for 96 h. Cell-free supernatant was collected by centrifugation at 10,000 rpm for 15 min at 4 °C and were used for extraction of biosurfactant compounds. After acid precipitation with solvent extraction, the obtained yellow-colored crude biosurfactant was filtered and evaporated to dryness in a vacuum evaporator (Yamato DC 400). The compounds were further separated by reversed phase high-performance liquid chromatography (HPLC) with SPD-20A/20AV UV detector system (Shimadzu HPLC LC-2010AHT) and C18 column (4.6 × 250 mm). Surface active compound from the column fractions was determined by thin-layer chromatography (TLC) with the solvent system butanol:acetic acid:water (4:3:2). The purified compound was mixed with potassium bromide and dried, which was then subjected to Fourier-transformed infrared (FT-IR) spectrophotometer analysis (Perkin Elmer 580 B, USA), at a spectral range 5,000–400/cm using an intuitive software interface of Spectrum 10™ to collect the spectral data. ^1^H NMR spectra were obtained by dissolving the purified lipopeptide in deuteron chloroform (CDCl_3_) at a concentration of 10 mg/ml and analyzed on a Bruker JNM-A500 spectrometer (Bruker BioSpin AG, Switzerland) at 400 MHz.

## Results and discussion

### Isolation and screening of biosurfactant producers

The survival of bacterial endosymbionts associated with marine sponges is attributed to massive stress caused by the vibrant change of surrounding sea water (Selvin et al. 2010). This implies that they have developed various structural and physiological changes, which permit them to compensate the effects of the adverse conditions. Moreover, metabolites from these sponge-associated microorganisms are characterized by well-known features, such as salt tolerance, hyper-thermo stability, barophilicity and adaptability to cold, all related to their habitat. However, scientific literature related to biosurfactant production from sponge-associated bacteria is scanty. Hence, an attempt was made in this study to explore the biosurfactant producing potential of marine sponge-associated bacteria. Of the various media used for culturing the sponge isolates, colonies were most abundant on MSA (2.8 × 10^5^ CFU/cm^3^ of sponge tissue) with lowest quantities on nutrient agar (1.14 CFU/cm^3^ of sponge tissue). In general, the addition of sponge extract and raffinose to MSA favored the growth of more isolates than other culture media used in the study. 101 sponge-associated bacteria were isolated on different media used in the present study. Among them 76 isolates showed positive results for at least one of the biosurfactant screening tests and 29 showed positive for the all screening assays. Whereas 68 and 33 isolates were found to be hemolytic and non-hemolytic, respectively. Among these 33 non-hemolytic isolates, 7 isolates were identified as biosurfactant producers. Based on the biosurfactant production potential, isolates MB-7, MB-I9, MB-30 and MB-101 were selected for further study. Biosurfactant production ability of these four isolates is listed in Table 1.

**Table 1:** Biosurfactant screening of selected four bacteria isolated from marine sponge *C. diffusa*

S. No	Screening test	MB-101	MB-7	MB-30	MB-I9
1.	Lipase activity (cm)	1.3 ± 0.04	0.9 ± 0.3	1.2 ± 0.05	0.8 ± 0.2
2.	Hemolytic activity	+	+	+	−
3.	Blue agar method	−	−	+	+
4.	Oil displacement (mm)	11 ± 0.2	9 ± 0.3	10 ± 0.2	10 ± 0.1
5.	Drop collapsing test	+	+	+	+
6.	Glass tilted method	+	+	+	+
7.	Surface tension (mN/m)	28 ± 0.6	28.8 ± 0.5	29 ± 0.45	29 ± 0.23
8.	Hydrophobicity (%) in stationary phase culture	63.2 ± 0.34	65.9 ± 0.72	79.2 ± 0.54	78.4 ± 0.37

+ Positive, − Negative

Since biosurfactants were heterogeneous in nature, it was very important to perform different screening methods. Therefore, a range of screening tests included to identify potential biosurfactant producers. Lipase acts on water–oil surfaces and, therefore, was suggested as one of the preliminary screening methods for biosurfactant production (Kokare et al. 2007). Production of biosurfactant (surfactin) by *B. subtilis* caused the red blood cells to lysis and had been widely used for preliminary screening test for biosurfactant production (Bernheimer and Avigad 1970). Surface tension reduction in liquid–liquid interface leads to complete spreading of liquid drop over the surface of oil (Youssef et al. 2004). Hence, the drop collapsing and oil displacement tests are considered as the easiest and effective method to screen biosurfactant producers. However, these techniques are not correlated to surface tension reduction to confirm its reliability (Youssef et al. 2004). The measurement of surface tension is an important criterion for selection of potential biosurfactant producers. The four biosurfactant producers were tested for this property and all of them were found to have potent surface tension reduction capability. The minimum surface tension reduction of all four culture broths (Zobell marine broth supplemented with 1 % glucose) were observed after 36 h of incubation (Table 1), during their exponential growth phase and the values were constant up to 96 h of incubation. This suggests that the biosurfactant concentration reached sufficient for micelle formation and beyond that constant surface tension was observed. Comparison of these results with previously reported biosurfactants (Shavandi et al. 2011; Joshi and Desai 2013) indicates that the biosurfactants from marine sponge-associated bacteria may act as potent biological surface active agents.

### Emulsification activity

**Fig. 1:**
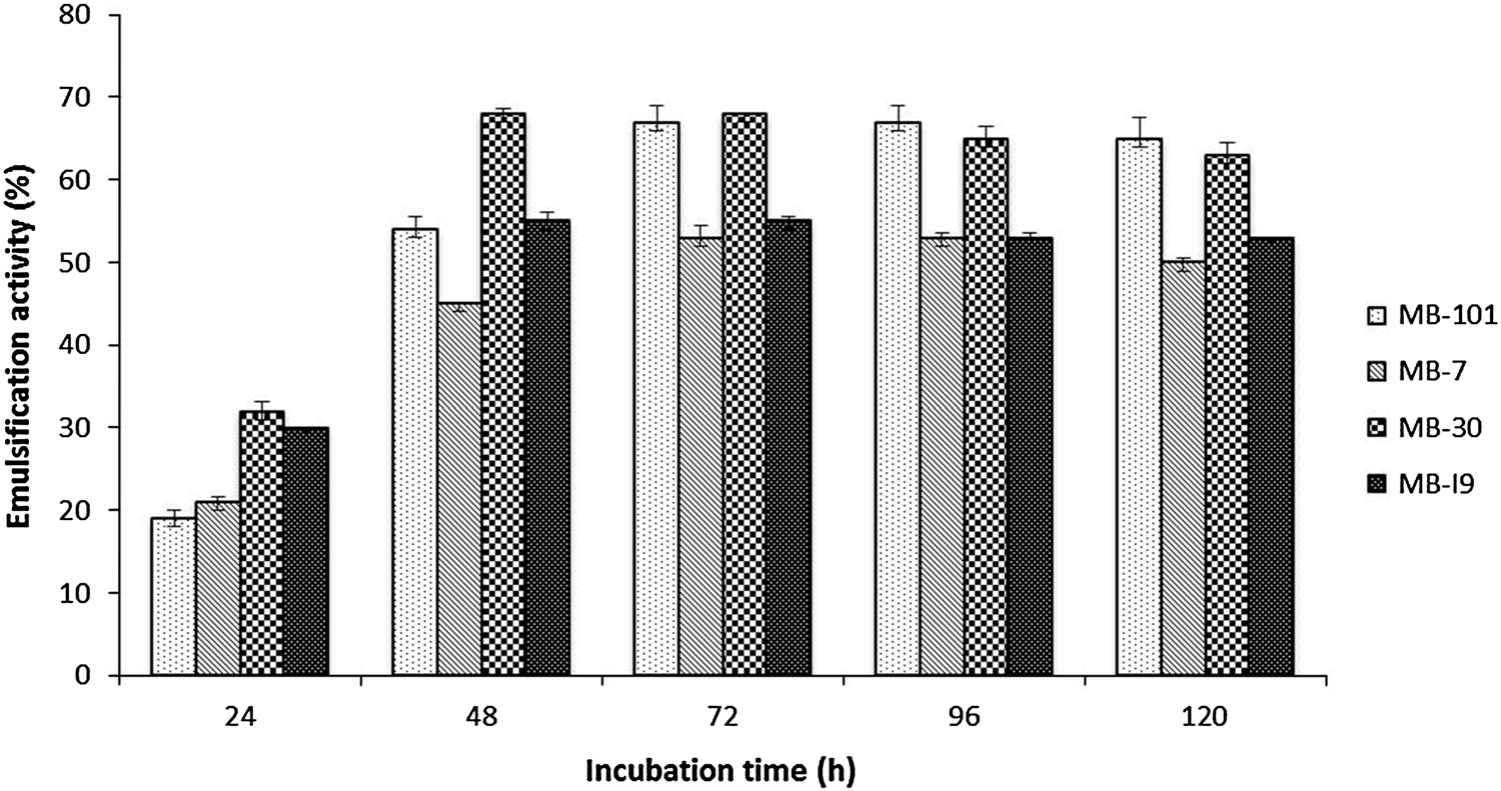
Emulsification activity of sponge isolated bacteria with different incubation time (h). The *error bars* denote SD values of three independent experiments

Emulsification activity of cell-free supernatant of four isolates grown in Zobell marine broth supplemented with 1 % glucose is presented in Fig. 1. Isolates MB-30 and MB-I9 were found to produce highest emulsification after 48 h of incubation, whereas isolates MB-7 and MB-101 produced highest emulsification after 72 h of incubation suggesting that the secondary metabolites such as biosurfactants are secreted during stationary phase of growth. A gradual increase in emulsification activity was observed with the concomitant increase of biomass concentration (data not shown) and it evidenced the growth-associated pattern of biosurfactant production. The positive correlation between biomass and biosurfactant concentration was observed in biosurfactant production by *Rhodococcus* sp. and *B. subtilis* 20B (Shavandi et al. 2011; Joshi et al. 2008). The emulsification activities of four isolates grown on glucose supplemented medium were found to be highest than earlier reported studies and the formed emulsions with kerosene were stable for 1 month at room temperature without the droplets coalescence (Gutierrez et al. 2007; Shavandi et al. 2011). In addition to surface activity, better emulsification activity was considered as vital for biosurfactants to be promising in various industrial and environmental applications.

### BATH assay

Cell hydrophobicity was used as one of the screening methods of biosurfactant production, because molecules with low surface energy easily attach to hydrophobic surfaces (Mozes and Rouxhet 1987) and it was measured by BATH assay. BATH assay can also be helpful for analyzing hydrocarbon utilization and degradation potential of biosurfactant producers. Isolation of hydrocarbon degrading bacteria is of great interest for the preservation of natural environment by reducing the amount of oil-related contaminants. It was found that during the initial stationary growth phase of four isolates, the increase in cell hydrophobicity ranged from 63.2 to 79.2 % (Table 1), suggesting the ability of the four bacterial isolates to adhere to hydrocarbon and produce biosurfactant. Literature reveals that bacterium with highest cell hydrophobicity shows more affinity towards hydrophobic substrates and thus considered as potent biosurfactant producers (Volchenko et al. 2007). The comparison of the hydrophobicity in the hydrocarbon (*n*-hexadecane): surfactant system indicated that the hydrophobicity of isolate MB-30 was found to be higher (79.2 %) than other three isolates. Hence isolate MB-30 may be utilized for enhancing biodegradation of hydrocarbon in oil-contaminated area. Isolation of halophilic/halotolerant microorganisms such as isolate MB-30 is environmentally significant since they accelerate removal of oils from polluted saline environments by virtue of their biosurfactant production ability (Margesin and Schinner 2001). Reports suggest use of biosurfactants in hydrocarbon pollution control in closed marine biotopes as well as in open coastal areas (Banat et al. 2000).

### Biochemical and molecular characterization of biosurfactant producers

**Fig. 2:**
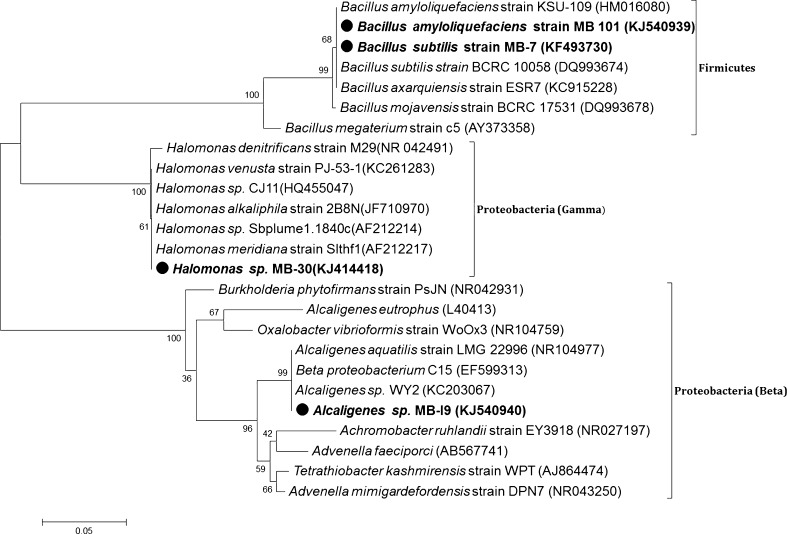
Evolutionary relationship of sponge isolated bacteria with other related species. *Numbers* at nodes indicate levels of bootstrap support (%) based on a neighbor-joining analysis of 1,000 re-sampled datasets

The morphological, physiological and biochemical characterizations of four selected isolates are listed in Table 2. 16S rRNA sequencing is a powerful tool for rapid identification and phylogenetic analysis of bacterial species. The isolates MB-7 and MB-101 showed unique branching from other nearest *Bacillus* species and were found to be closely related to *B. subtilis* (99 % similarity) and *B. amyloliquefaciens* (99 % similarity), respectively. The isolates MB-I9 and MB-30 were enrolled into a cluster-containing *Alcaligenes* sp. and *Halomonas* sp., respectively. Based on the biochemical characterization and phylogenetic analysis, the four isolates were designated as *B. subtilis* MB-7, *B. amyloliquefaciens* MB-101, *Halomonas* sp. MB-30 and *Alcaligenes* sp. MB-I9 (Fig. 2). The sequence data of the selected biosurfactant producers were assigned with the GenBank accession numbers KF493730 (*B. subtilis* MB-7), KJ540940 (*Alcaligenes* sp. MB-I9), KJ414418 (*Halomonas* sp. MB-30) and KJ540939 (*B. amyloliquefaciens* MB-101), respectively.

**Table 2:** Biochemical identification of biosurfactant producing bacterial isolates from marine sponge

Test	MB-101	MB-7	MB-30	MB-I9
Gram staining	+	+	−	−
Motility	+	+	+	+
Cell shape	Rods	Rods	Small rods	Small rods
Indole	+	−	−	−
Methyl red	−	+	+	+
VP	−	+	+	+
Citrate	+	+	+	+
Oxidase	+	+	−	+
Catalase	+	+	+	+
Hydrolytic activity				
Starch	+	+	+	−
Casein	+	+	−	−
Tributyrin	+	+	+	+
Urea	−	−	+	−
Fermentation test				
Glucose	+	+	+	−
d-galactose	−	−	−	W
Mannitol	W	+	+	−
Sucrose	+	+	_+_	−

+ Positive, − Negative, *W* weakly positive

The diverse bacterial phyla associated with most sponges are reported to be α, β, γ, δ and ε Proteobacteria and Chloroflexi (Hentschel et al. 2002; Taylor et al. 2007). In this study, the sponge isolates MB-I9 and MB-30 were grouped into proteobacteria and the other two isolates including MB-7 and MB-101 were under firmicutes. Most of the *Bacillus* sp. isolated from marine invertebrates are highly resistant to extremes of pH, temperature, irradiation and desiccation. The spore-forming ability of *Bacillus* sp. facilitates easy adaptation to diverse environmental conditions. *Bacillus* sp. is the most efficient producer of different lipopeptide structures such as surfactin, lichenysin, fengycin, bacillomycin and iturin (Vater et al. 2002). In the present investigation, *B. subtilis* MB-7 was found to have salt tolerance up to 7 %. The potent strain *Alcaligenes* sp. MB-I9 was non-hemolytic biosurfactant producer. Non-hemolytic property of biosurfactant has been reported earlier (Das et al. 2008). Most of the biosurfactant producing bacteria identified so far are mesophilic and hence more attention has been focused on biosurfactant production under extreme conditions (Cameotra and Makkar 1998). The rapid adjustment to changes in the external salt concentration makes bacterial forms isolated from marine environment potential candidates for bioprocessing.

### Statistical optimization for biosurfactant production

The biosurfactant production by sponge-associated *B. amloliquefaciens* has not been reported earlier. Thus, this study demonstrated the biosurfactant production potential of the strain MB-101 by submerged fermentation employed with statistical optimization. The variables/factors such as glycerol, peptone, ferrous sulfate, and incubation time showed significant effect on biosurfactant production as evident by the regression coefficient values (ANOVA) obtained after fractional factorial optimization (Online resource 3). Based on the fractional factorial design, the optimum levels of following parameters such as pH at 7, temperature at 37° C salt concentration at 2 % and 96 h of incubation increased the biosurfactant yield, and hence these parameter levels were maintained for further optimization with CCD. A CCD was developed to understand the interactions among most significant independent variables (glycerol, peptone, ferrous sulfate, and incubation time) and their effect on biosurfactant production. The generated CCD experimental design with their response values is listed in online resource 4. The *F* test for an ANOVA was developed to understand the statistical significance and reliability of the regression model (Table 3).

**Table 3:** Analysis of variance (ANOVA) of the central composite design optimization for the production of biosurfactant by MB-101

Source	Sum of squares	*df*	Mean square	*F* value	*p* value prob > *F*
Model	21.32	14	1.52	67.58	<0.0001**
A-glycerol	2.86	1	2.86	127.10	<0.0001**
B-peptone	1.11	1	1.11	49.43	<0.0001**
C-ferrous sulfate	0.033	1	0.033	0.033	0.2449
D-incubation time	0.64	1	0.64	28.27	<0.0001**
*AB*	0.014	1	0.014	0.61	0.4459
*AC*	0.032	1	0.032	1.40	0.2554
*AD*	0.22	1	0.22	9.91	0.0066*
*BC*	0.31	1	0.31	13.80	0.0021*
*BD*	0.44	1	0.44	19.48	0.0005**
*CD*	0.28	1	0.28	12.35	0.0031*
*A* ^2^	11.39	1	11.39	505.67	<0.0001**
*B* ^2^	4.80	1	4.80	212.95	<0.0001**
*C* ^2^	1.51	1	1.51	66.94	<0.0001**
*D* ^2^	2.48	1	2.48	110.10	<0.0001**
Residual	0.34	15	0.023		
Lack of fit	0.34	10	0.034		

*R*-Squared −0.9844; Adj *R*-squared 0.9698

** More significant, * significant

**Fig. 3:**
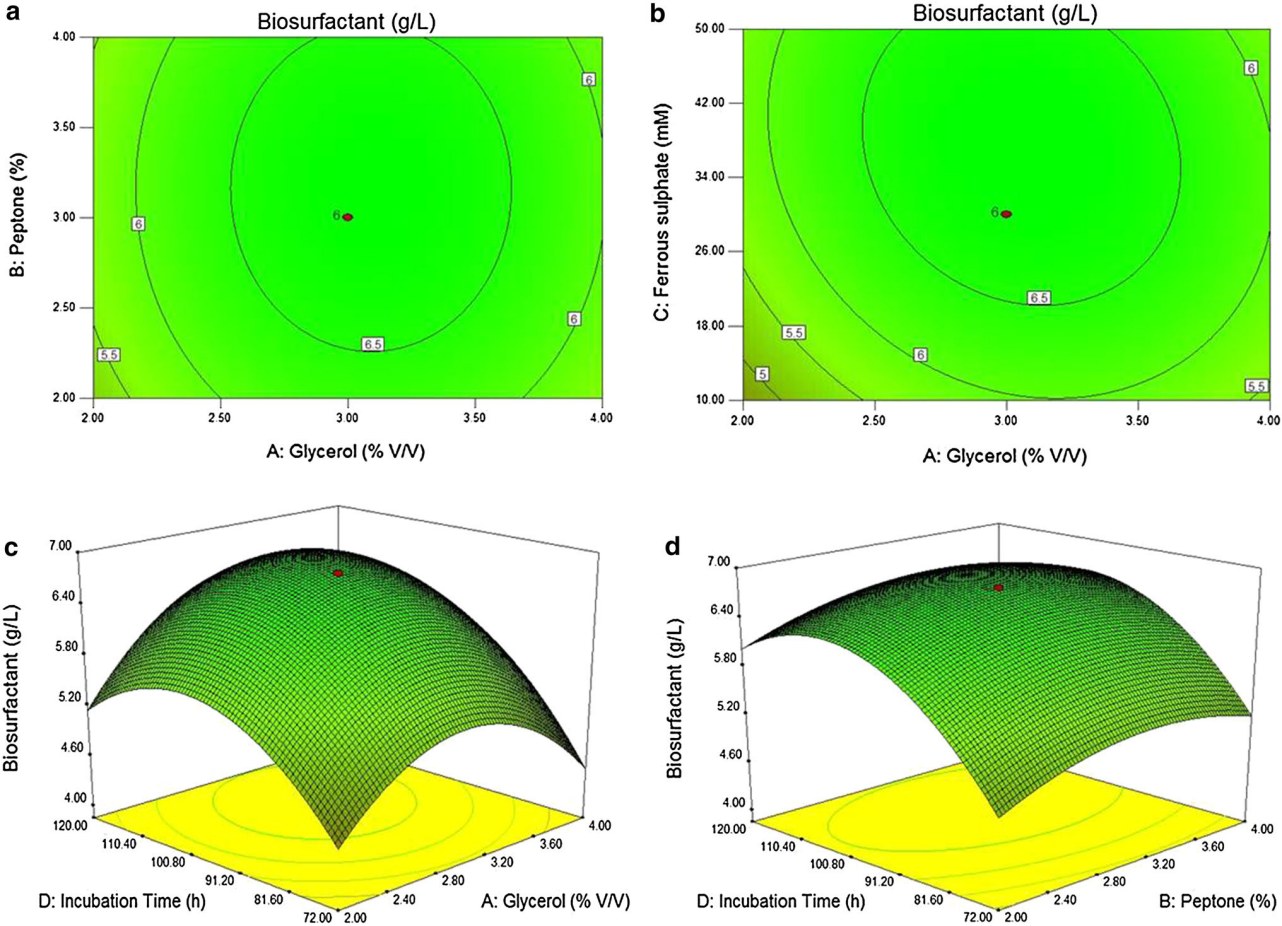
**a** Contour plot of interactive effect of glycerol: peptone, **b** glycerol: ferrous sulphate, **c** 3D response surface graph of interactive effect of glycerol: incubation time and **d** peptone: incubation time on lipopeptide biosurfactant production by *B. amyloliquefaciens* MB-101, while other two factors were held at fixed levels

ANOVA result suggested that, all the model terms except *AB* and *AC*, in the examined range were found to be significant (*p* > 0.0001). The fit value, termed *R* ^*2*^ (determinant coefficient), was calculated to be 0.9844 for biosurfactant production by MB-101 suggesting that 98.44 % of the variability in the response could be explained by the polynomial model and hence the final equation in terms of coded factor may be written as follows:

1 $$\begin{gathered} {\text{Biosurfactant }}\left( {{\text{g}}/{\text{l}}} \right) = + 6.76 \, + 0.17* \, A + 0.11* \, B + 0.34* \, C + 0.52* \, \hfill \\ D - 3.125{\text{E}} - 003*AB - 0.18* \, AC + 0.21*AD - \, 0.073*BC - 0.033*BD \hfill \\ + 0.46*CD - 0.91* \, A^{ 2} - 0.34* \, B^{ 2} - 0.46*C^{ 2} - 0.84* \, D^{ 2} \hfill \\ \end{gathered}$$

where, *Y* was the response of biosurfactant yield and *A*, *B*, *C*, and *D* were the coded terms for the independent test variables of glycerol, peptone, ferrous sulphate and incubation time, respectively. Contour plots and three-dimensional response surface of significant factors (*AB*, *AC*, *AD* and *BD*) interaction on biosurfactant production generated by the CCD model are shown in Fig. 3, whereas the other two factors were kept constant. The calculated *F* value implied that the model was significant at *p* < 0.0001 and there was 0.01 % chance of Model *F* value due to noise. The predicted *R* ^*2*^ of 0.9844 was reasonable in agreement with the Adj *R* ^*2*^ of 0.9698. Adequate precision measures the signal to noise ratio and, therefore, the ratio 67.58 indicates an adequate signal and thus this model could be used to navigate the design space.

The experimental and the predicted values of most points were obtained near to the fit line (Online resource 5). Thus, the observed and predicted values are best explained by this model for maximum biosurfactant production. Based on Fig. 3 and Eq. (1), the CCD model predicted a maximum biosurfactant concentration of 6.76 g/l, with optimal concentration values of 2.83 % for glycerol, 2.65 % for peptone, 20.11 mM for ferrous sulfate and 74 h of incubation time. The carbon source glycerol increased the biosurfactant production by *B*. *amlyoliquefaciens* MB-101 up to 6.76 g/l with other optimal process parameters. It was found that from the preliminary screening of carbon substrate, glycerol showed highest biosurfactant production followed by sucrose and glucose than the selected cheapest sources of tamarind kernel powder and corn powder and also the concentration of 2–3 % range of glycerol was found to be enough for highest biosurfactant production by *B.* *amlyoliquefaciens* MB-101 strain. Literature evidenced that the readily available hydrophilic carbon sources such as glycerol, glucose and mannitol are considered as the best source for biosurfactant production (Das et al. 2009). The organic nitrogen source peptone enhanced the biosurfactant production than selected inorganic nitrogen sources. Production of biosurfactant by *B. amyloliquefaciens* MB-101 was increased by the addition of FeSO_4_ below the range of 50 mM concentration. Wei and Chu (1998) suggested that the iron concentration of micromolar to the millimolar level highly enhances the biosurfactant production by *B. subtilis* ATCC 21332. It was established that the iron was the key metal for the production of secondary metabolites by *Bacillus* sp. and actinobacteria without having an effect on cell growth (Cooper et al. 1981; Kiran et al. 2014a). In this study, the production of biosurfactant from *B. amyloliquefaciens* MB-101 was significantly elevated up to 6.76 g/l and the yield was 3.48 fold higher than that of non-optimized normal media. Statistical approaches aid in the formulation of production medium of biosurfactant and may be crucial to enhance the quantity of the product (Kiran et al. 2010). Therefore, the *B. amyloliquefaciens* MB-101 can be used as potential strain for the commercial production of biosurfactant under submerged fermentation.

### Structural characterization

**Fig. 4:**
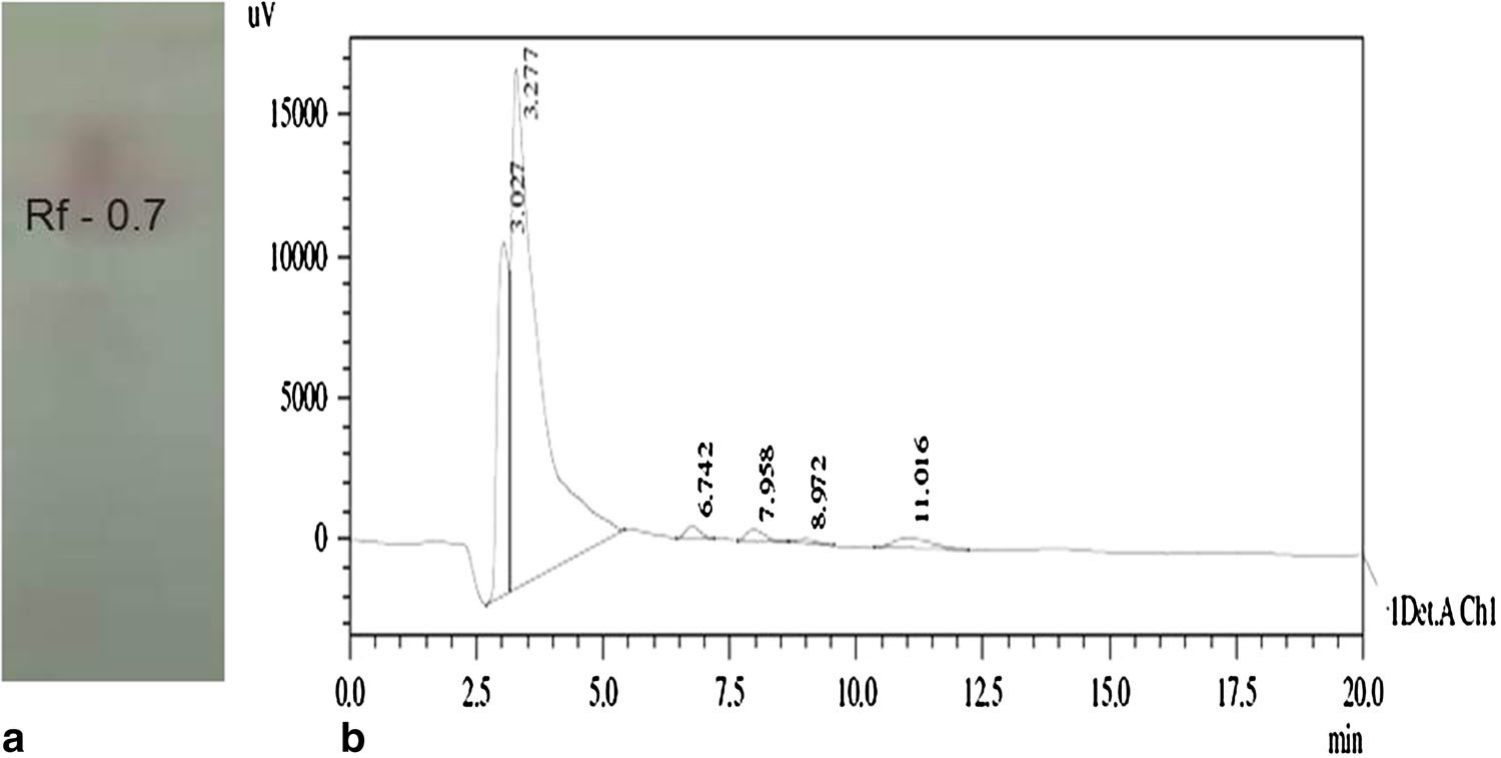
**a** TLC and **b** HPLC profile of biosurfactant extracted from *B. amyloliquefaciens* MB-101

**Fig. 5:**
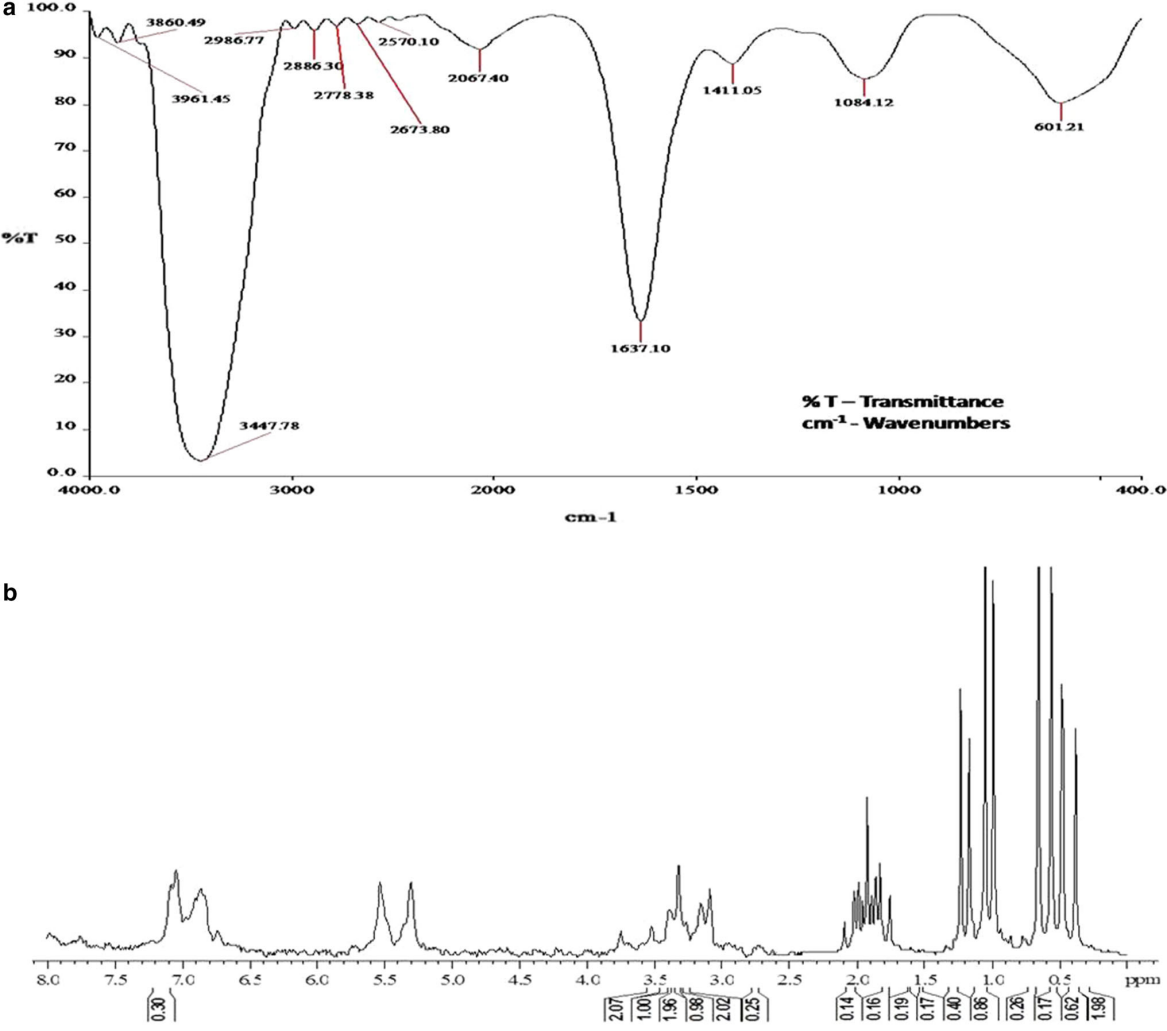
**a** FTIR absorption spectra of biosurfactant produced by *B. amyloliquefaciens* MB-101 and **b** ^1^H NMR spectrum of MB-101 biosurfactant (CDCl_3_ at 25 °C)

The biosurfactant compound extracted from *B*. *amyloliquefaciens* MB-101 contained 1.8 % of carbohydrate, 16.3 % of protein and 25.6 % of lipid. The presence of lipid and peptide moieties in the biosurfactant molecule was confirmed by TLC analysis with the existence of single spot with the *R*_f_ value of 0.7 in lipopeptide-specific solvent system (Fig. 4a). Moreover, the HPLC showed the resolving pattern of partially purified biosurfactant and the compound eluted at 3.277 min exhibited highest oil displacement activity and emulsification activity (Fig. 4b). The purified biosurfactant was characterized by FT-IR analysis and the spectra revealed the presence of aliphatic and peptide moieties (Fig. 5a). The absorbance band at 3,447 was assumed to form as a result of N–H stretching mode of peptide portion, at 1,637 corresponds to the N–H primary amine (NH bend) and at 1,084 indicates the C–N stretching of aliphatic amines. In addition, the presence of absorbance bands at 2,936 and 2,886 indicates the asymmetrical stretching of CH_2_ and symmetrical stretching of CH_2_ of methylene groups of aliphatic side chain, respectively. The overall absorbance band obtained from the FT-IR spectrum of biosurfactant obtained from *B*. *amyloliquefaciens* MB-101 consistent with the reported lipopeptide biosurfactant structures (Joshi et al. 2008; Saimmai et al. 2013). In ^1^H-NMR spectra, the amide protons of amino acids of biosurfactant structure appeared at δ 6.8–7.9 ppm. The peptide signal appearing at δ 2.8–3.8 represents the methine and methylene protons of amino acids. The proton signals at δ 0.3–1.4 and 1.7–2.4 correspond to the fatty acid side chains of biosurfactant compound. From the overall chemical shifts appearing in the ^1^H-NMR (Fig. 5b), the produced biosurfactant from *B. amyloliquefaciens* MB-101 confirmed the lipopeptide nature of biosurfactant (Morikawa et al. 1993; Yakimov et al. 1995). Zhao et al. (2013) reported that the *B. amyloliquefaciens* Q-426 strain produced antifungal lipopeptide with excellent biosurfactant properties. The strain *B. amyloliquefaciens* LSC04 produced a novel lipopeptide, designated as fengycin S, having highest surface activity and the strain can itself degrade crude oil efficiently (Sang-Cheol et al. 2010). *Bacillus* sp. has been the most efficient producer of different lipopeptide biosurfactants and has gained utmost importance because of its unique structure and biological activities.

## Conclusions

The excellent surfactant tension reducing characteristics and highest oil displacement ability of lipopeptide biosurfactant of *B. amyloliquefaciens* MB-101 imply the possibility of using bioremediation process in the marine environment. The range of low- and high-molecular weight biosurfactants produced by microorganisms, lipopeptides’ groups generally comes under low molecular weight biosurfactants that are more effectively reduce surface and interfacial tension. An extensive study on the peptide moieties and protein component of biosurfactants with the help of genomics, proteomics and metabolomics tools will undoubtedly provide an insight into the detailed structure and specific function of the biosurfactant, which can thus be further modified or improved for wide applicability.

## Electronic supplementary material

Below is the link to the electronic supplementary material.

Supplementary material 1 (DOCX 72 kb)
