# Supplementary material for: Biosurfactant production from marine bacteria associated with sponge Callyspongia diffusa
Source: 3 Biotech. 2014 Aug 13;5(4):443–54. doi: 10.1007/s13205-014-0242-9 (PMC4522725; doi:10.1007/s13205-014-0242-9)

3 Biotech

Online resource materials

**Assessment of biosurfactant production from marine bacteria associated with sponge *Callyspongia diffusa* collected from southern coast of India**

Asha Dhasayan^1^, Joseph Selvin^2*^and Seghal Kiran^3^

^1^Department of Microbiology, School of Life Sciences, Bharathidasan University, Tiruchirappalli-620024, Tamil Nadu, India

^2^Center for Microbiology, Pondicherry University, Puducherry-605014, Tamil Nadu, India

^3^Department of Food Science and Technology, Pondicherry University, Puducherry-605014, Tamil Nadu, India

E-Mails: [ashadhasayan@gmail.com](mailto:ashadhasayan@gmail.com) (DA), [seghalkiran@gmail.com](mailto:seghalkiran@gmail.com) (SK)

*Correspondence:

**Dr. Joseph Selvin**,

Centre for Microbiology, Pondicherry University, R.V. Nagar, Kalapet,

Puducherry - 605 014, India.

Tel.: +91 9944263367; Fax.: +91413 – 2655734

E-mail address: josephselvinss@gmail.com, jselvin.mib@pondiuni.edu.in (J. Selvin)

# Online resource 1 Factors and range selected for Fractional factorial statistical experimental design (2^9-^*^5^*)

| Name | Units | Low | High |
| --- | --- | --- | --- |
| Glycerol | % (v/v) | 1 | 2 |
| Soyabean Powder | % | 1 | 2 |
| Peptone | % | 1 | 2 |
| Ferrous sulphate | mM | 50 | 100 |
| Ferric chloride | mM | 50 | 100 |
| Magnesium Chloride | mM | 50 | 100 |
| Incubation Time | h | 72 | 96 |
| pH |  | 6 | 7 |
| Temperature | °C | 28 | 37 |

**Online resource 2** Distribution of factors according to the 2-Level Factorial design (coded values) and the response values for different media components evaluation on biosurfactant production by MB-101

| **Run** | A | B | C | D | E | F | G | H | J | X |
| --- | --- | --- | --- | --- | --- | --- | --- | --- | --- | --- |
| 1 | -1 | 1 | 1 | 1 | -1 | 1 | -1 | -1 | -1 | 1.83 |
| 2 | -1 | 1 | -1 | 1 | 1 | -1 | 1 | -1 | 1 | 1.96 |
| 3 | -1 | -1 | 1 | 1 | 1 | 1 | 1 | -1 | -1 | 2.69 |
| 4 | 1 | -1 | -1 | 1 | 1 | 1 | -1 | -1 | 1 | 1.79 |
| 5 | -1 | -1 | -1 | 1 | -1 | 1 | 1 | 1 | -1 | 1.58 |
| 6 | 1 | -1 | 1 | 1 | -1 | -1 | 1 | -1 | -1 | 2.34 |
| 7 | 1 | 1 | -1 | -1 | -1 | 1 | 1 | 1 | 1 | 2.99 |
| 8 | 1 | 1 | 1 | -1 | 1 | -1 | -1 | -1 | -1 | 2.6 |
| 9 | 1 | -1 | 1 | -1 | -1 | 1 | -1 | 1 | 1 | 2.78 |
| 10 | -1 | -1 | -1 | -1 | -1 | -1 | -1 | -1 | 1 | 1.86 |
| 11 | 1 | 1 | 1 | 1 | 1 | 1 | 1 | 1 | 1 | 2.75 |
| 12 | 1 | -1 | -1 | -1 | 1 | -1 | 1 | 1 | -1 | 2.44 |
| 13 | -1 | 1 | -1 | -1 | 1 | 1 | -1 | 1 | -1 | 1.45 |
| 14 | -1 | 1 | 1 | -1 | -1 | -1 | 1 | 1 | 1 | 1.94 |
| 15 | -1 | -1 | -1 | 1 | 1 | -1 | -1 | 1 | 1 | 1.82 |
| 16 | 1 | 1 | -1 | 1 | -1 | -1 | -1 | 1 | -1 | 2.25 |

^A:Glycerol % (v/v), B:Soyabean Powder (%),C: Peptone (%),D: Ferrous sulphate (mM), E:Ferric chloride (mM),^

^F:Magnesium Chloride (mM), G:Incubation Time (h), H:pH, J:Temperature (°C), Biosurfactant (g/L).^

**Online resource 3** Analysis of Variance (ANOVA) for 2-Level Factorial design on biosurfactant production by *Bacillus amyloliquefaciens* MB-101

| Source | Sum of Squares | df | Mean Square | F Value | p-value  Prob > F |
| --- | --- | --- | --- | --- | --- |
| Model | 3.14 | 10 | 0.31 | 6.64 | 0.0248 |
| A-Glycerol | *1.45* | *1* | *1.45* | *30.55* | *0.0027** |
| *B-Soyabean Powder* | *0.014* | *1* | *0.014* | *0.29* | *0.6123* |
| C-Peptone | *0.37* | *1* | *0.37* | *7.80* | *0.0383** |
| D-Ferrous sulphate | *0.37* | *1* | *0.37* | *7.80* | *0.0383** |
| E-Ferric chloride | *3.062E-004* | *1* | *3.062E-004* | *6.471E-003* | *0.9390* |
| *F-Magnesium Chloride* | *0.026* | *1* | *0.026* | *0.56* | *0.4887* |
| *G-Incubation Time* | *0.33* | *1* | *0.33* | *7.05* | *0.0452** |
| *H-pH* | *0.069* | *1* | *0.069* | *1.46* | *0.2815* |
| *AB* | *0.25* | *1* | *0.25* | *5.34* | *0.0689* |
| *AH* | *0.26* | *1* | *0.26* | *5.55* | *0.0651* |
| Residual | 0.24 | 5 | 0.047 |  |  |
| Cor Total | 3.38 | 15 |  |  |  |

^R-Squared- 0.9300; Adj R-Squared- 0.7899,^ *^*^* ^significant^

**Online resource 4** Distribution of factors according to CCD and the response values for different media components evaluation of biosurfactant production (MB-101)

|  | Factor 1 | Factor 2 | Factor 3 | Factor 4 | Response 1 |
| --- | --- | --- | --- | --- | --- |
| Run | A:Glycerol  % (v/v) | B:Peptone  (%) | C:Ferrous  Sulphate (mM) | D:Incubation  Time(h) | Biosurfactant  (g/L) |
| 1 | 3.00 | 1.00 | 30.00 | 96.00 | 5.18 |
| 2 | 3.00 | 3.00 | 30.00 | 96.00 | 6.76 |
| 3 | 2.00 | 2.00 | 50.00 | 72.00 | 3.90 |
| 4 | 3.00 | 3.00 | 30.00 | 96.00 | 6.76 |
| 5 | 3.00 | 3.00 | -10.00 | 96.00 | 4.16 |
| 6 | 4.00 | 2.00 | 50.00 | 120.00 | 5.80 |
| 7 | 2.00 | 2.00 | 50.00 | 120.00 | 5.24 |
| 8 | 4.00 | 4.00 | 10.00 | 72.00 | 4.37 |
| 9 | 3.00 | 5.00 | 30.00 | 96.00 | 5.49 |
| 10 | 4.00 | 4.00 | 10.00 | 120.00 | 4.47 |
| 11 | 3.00 | 3.00 | 30.00 | 96.00 | 6.76 |
| 12 | 2.00 | 4.00 | 10.00 | 120.00 | 3.73 |
| 13 | 3.00 | 3.00 | 30.00 | 48.00 | 2.18 |
| 14 | 3.00 | 3.00 | 30.00 | 144.00 | 4.51 |
| 15 | 4.00 | 4.00 | 50.00 | 72.00 | 3.60 |
| 16 | 2.00 | 4.00 | 10.00 | 72.00 | 3.85 |
| 17 | 2.00 | 2.00 | 10.00 | 120.00 | 3.08 |
| 18 | 3.00 | 3.00 | 30.00 | 96.00 | 6.76 |
| 19 | 3.00 | 3.00 | 70.00 | 96.00 | 5.51 |
| 20 | 2.00 | 2.00 | 10.00 | 72.00 | 3.50 |
| 21 | 3.00 | 3.00 | 30.00 | 96.00 | 6.76 |
| 22 | 2.00 | 4.00 | 50.00 | 72.00 | 3.84 |
| 23 | 2.00 | 4.00 | 50.00 | 120.00 | 5.34 |
| 24 | 3.00 | 3.00 | 30.00 | 96.00 | 6.76 |
| 25 | 4.00 | 2.00 | 10.00 | 72.00 | 3.77 |
| 26 | 4.00 | 2.00 | 50.00 | 72.00 | 3.21 |
| 27 | 4.00 | 4.00 | 50.00 | 120.00 | 5.80 |
| 28 | 1.00 | 3.00 | 30.00 | 96.00 | 2.81 |
| 29 | 4.00 | 2.00 | 10.00 | 120.00 | 4.47 |
| 30 | 5.00 | 3.00 | 30.00 | 96.00 | 3.29 |

**Online resource 5** Plots of predicted vs. actual values of biosurfactant concentration (g L-1) produced by *Bacillus amyloliquefaciens* MB-101 during optimization of fermentation in CCD.


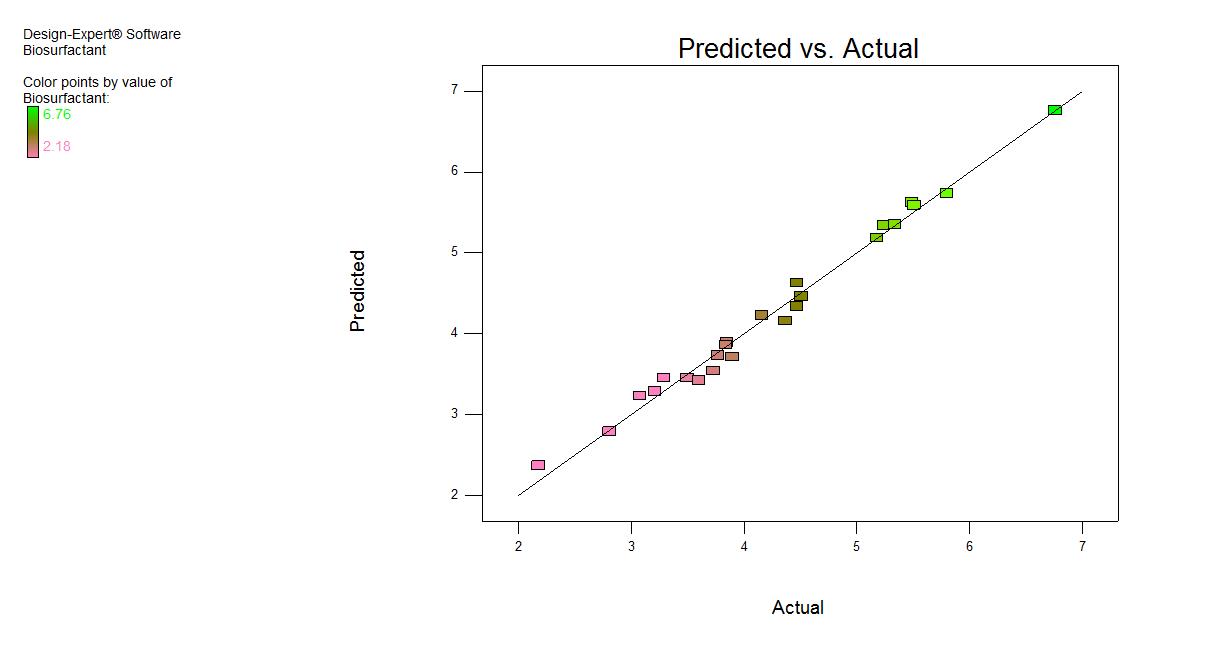

Supplement: Supplementary file 1 — Supplementary material 1 (DOCX 72 kb) [file 13205_2014_242_MOESM1_ESM.docx]
